# The Superoxide Dismutase Family in Balloon Flower (*Platycodon grandiflorus*): Phylogenetic Relationships, Structural Characteristics, and Expression Patterns

**DOI:** 10.3390/cimb47050351

**Published:** 2025-05-12

**Authors:** Tae Kyung Hyun

**Affiliations:** Department of Industrial Plant Science and Technology, College of Agriculture, Life and Environment Sciences, Chungbuk National University, Cheongju 28644, Republic of Korea; taekyung7708@chungbuk.ac.kr; Tel.: +82-43-261-2520

**Keywords:** antioxidant, *Platycodon grandiflorus*, superoxide dismutase

## Abstract

Superoxide dismutases (SODs) are essential antioxidant enzymes that protect plant cells from oxidative stress, thereby preserving cellular integrity. This study presents a comprehensive genome-wide analysis of the SOD gene family in *Platycodon grandiflorus*, identifying seven genes classified into three distinct groups based on phylogenetic relationships. Detailed bioinformatics analyses revealed variations in structural characteristics and physicochemical properties. PlgSODs were predicted to localize primarily to the chloroplast and mitochondria. Tissue-specific expression patterns indicate that PlgSOD genes play important roles in plant growth and development. Furthermore, promoter analysis identified several potential transcription factors (TFs), including members of the B3, Dof, and MYB-related families, which are known for their involvement in stress responses. These TFs are likely to regulate multiple PlgSOD genes, suggesting a coordinated transcriptional regulation mechanism under specific physiological or stress conditions. Taken together, these findings provide valuable insights into the functional roles of SODs in *P. grandiflorus* and lay the groundwork for future genetic and biotechnological strategies aimed at enhancing stress tolerance in this species.

## 1. Introduction

Plants continuously fine-tune their metabolic networks to maintain homeostasis in response to environmental fluctuations. Central to this process are three oxidative pathways—photosynthesis, photorespiration, and oxidative respiration—which are interconnected through shared redox mediators and antioxidant defense systems [1]. This dynamic interplay regulates reactive oxygen species (ROS) levels and maintains redox homeostasis, enabling plants to adapt to changing conditions while preserving cellular integrity and metabolic efficiency [2]. However, environmental stresses often lead to the excessive accumulation of ROS, including singlet oxygen, hydrogen peroxide (H_2_O_2_), and superoxide anions. While moderate ROS levels are essential for cellular signaling, excessive ROS causes oxidative damage, disrupts membrane integrity, and triggers cell death, ultimately reducing crop productivity [2]. Therefore, maintaining a delicate balance between ROS production and antioxidant defense is crucial for plant survival and stress adaptation.

Higher plants have evolved intricate antioxidant systems comprising both enzymatic components—including superoxide dismutase (SOD), catalase, ascorbate peroxidase, glutathione peroxidase, and peroxiredoxin—and non-enzymatic defense mechanisms mediated by phytochemicals [3]. Among these, SODs, a class of metalloenzymes, function as the first line of defense against oxidative stress. They catalyze the dismutation of superoxide anions into molecular oxygen (O_2_) and hydrogen peroxide (H_2_O_2_), thereby mitigating ROS and protecting cells from oxidative damage [4]. Genetic and molecular studies have highlighted the critical role of SODs in enhancing plant resilience to various environmental stresses [4,5,6,7]. Consequently, modulating SOD activity has emerged as a promising strategy for developing stress-tolerant crops.

*Platycodon grandiflorus* (2*n* = 18), a medicinally and agriculturally valuable herbaceous perennial known for its bell-shaped flowers, belongs to the Campanulaceae family [8]. Its roots are widely used in traditional medicine and as food additives due to their well-documented therapeutic properties, making it an economically important medicinal crop in Asia, particularly in China and Korea [9]. Despite its significance, a chromosome-scale reference genome for *P. grandiflorus* was only recently assembled using high-throughput chromosome conformation capture technology [10]. However, a formal nomenclature for PlgSOD genes has yet to be established.

The present study conducted a genome-wide analysis of the SOD gene family in *P. grandiflorus*, identifying seven PlgSOD genes. A comprehensive phylogenetic analysis incorporating SOD homologs from other species classified the PlgSODs into three distinct groups. These findings lay the foundation for future functional analyses of PlgSOD genes and provide valuable genetic resources for improving stress tolerance in *P. grandiflorus*.

## 2. Materials and Methods

### 2.1. Identification and Characterization of the PlgSOD Gene Family

To systematically identify members of the *PlgSOD* gene family, a genome-wide search was conducted using BLAST algorithms, with SOD protein sequences from *Arabidopsis thaliana* and *Oryza sativa* as query templates [10]. The putative *PlgSOD* genes were subjected to comprehensive bioinformatics analyses, including conserved domain detection, molecular weight estimation, isoelectric point (pI) calculation, instability index determination, grand average of hydropathicity (GRAVY) assessment, and subcellular localization prediction, following methodologies modified from Ahn et al. [11]. Phylogenetic classification was performed to categorize PlgSODs into distinct subgroups. Structural features, including exon–intron organization, were analyzed using the Gene Structure Display Server (GSDS 2.0; https://gsds.gao-lab.org/, accessed on 1 March 2025) by aligning mRNA sequences with their corresponding genomic counterparts. To investigate the regulatory landscape of *PlgSOD* expression, 1.5 kb upstream promoter sequences were retrieved and analyzed for potential transcription factor binding sites. The PlantRegMap database (https://plantregmap.gao-lab.org/, accessed on 1 March 2025) was used for transcriptional regulator prediction, applying a stringent statistical threshold (*p* ≤ 10^−5^) to ensure high-confidence results.

### 2.2. Analysis of Tissue-Specific Expression Patterns

The tissue-specific expression profiles of *PlgSOD* genes were examined using RNA-Seq datasets from eight distinct tissue types, retrieved from NCBI GenBank (SRR8712510–SRR8712517). Transcript abundance was quantified in fragments per kilobase of transcript per million mapped reads (FPKM) to normalise expression levels across samples, following analytical methodologies adapted from Kim et al. [8].

## 3. Results and Discussion

### 3.1. Identification of PlgSODs

The recently sequenced *P. grandiflorus* genome enabled the systematic identification of putative *SOD* genes in this species. A BLAST-based homology search was conducted against the genomic database using functionally characterized SOD sequences from *A. thaliana* and *O. sativa* as reference queries. To ensure accuracy, redundant sequences were removed through self-BLAST filtering, followed by manual curation. Using this approach, seven putative *SOD* genes were identified and designated *PlgSOD1* to *PlgSOD7*, exhibiting 58–80% sequence identity with SOD proteins from Arabidopsis (Table 1).

The physicochemical properties of the identified PlgSOD proteins—including molecular weight, isoelectric point (pI), GRAVY index, and subcellular localization, were predicted using ExPASy, ProtParam, and WoLF PSORT. The PlgSOD proteins ranged in length from 198 amino acids (PlgSOD7) to 321 amino acids (PlgSOD4), with corresponding molecular weights of 20.48 kDa and 33.88 kDa, respectively (Table 1), highlighting their structural diversity. The instability index, an indicator of protein stability [12], revealed that six of the seven PlgSOD proteins were predicted to be stable, whereas PlgSOD1 had an instability index exceeding 40, suggesting reduced stability (Table 1). The stability of stress-resistance proteins, including SODs, under diverse environmental conditions is crucial for effective plant stress tolerance. Therefore, enhancing protein stability could be a strategic approach to improving resilience by preserving enzymatic function under stress. Protein engineering techniques, such as site-directed mutagenesis, have been shown to enhance stability. For example, cysteine-to-alanine substitutions in Cu/Zn-SODs from *Bos taurus* (Cys6 to Ala) [13], *Danio rerio* (Cys7 to Ala) [14], *Xenopus laevis* (Cys 150 to Ala) [15], and *Potentilla atrosanguinea* (Cys95 to Ala) [16] have demonstrated improved protein robustness. Similarly, the activity and stability of Mn-SOD from Mus musculus were enhanced by substituting V140, E155, and E215 with L140, W155, and W215, respectively [17]. These examples suggest that similar targeted modifications may enhance the stability of PlgSOD proteins. Further structural and functional characterization may help identify PlgSOD-specific residues suitable for future mutagenesis studies.

GRAVY values, which indicate the hydropathic nature of a protein, are calculated as the sum of the hydropathy indices of all amino acids divided by the total number of residues. Lower GRAVY values indicate higher hydrophilicity and better solubility in aqueous environments, while higher values suggest increased hydrophobicity [18]. In the present study, three PlgSODs exhibited positive GRAVY values, whereas four PlgSODs displayed values below zero (Table 1), indicating that most PlgSOD proteins are hydrophilic.

**Figure 1:**
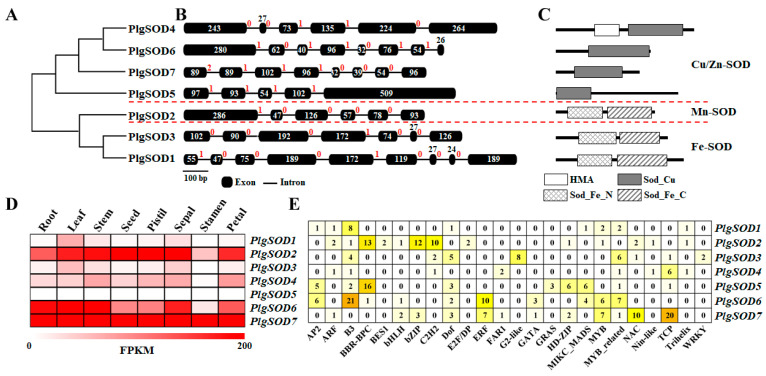
Genome-wide characterization of the SOD family in *Platycodon grandiflorus*. (**A**) Phylogenetic analysis, (**B**) gene structure, and (**C**) domain composition of PlgSODs. The phylogenetic tree was constructed using the neighbor-joining method in MEGA7. Exon lengths (in nucleotides) are indicated above the exons, while intron phases are denoted above the introns. Conserved domains were identified using the SMART program. (**D**) Tissue-specific expression patterns of *PlgSODs*, based on RNA-Seq analysis of eight different tissues, represented as fragments per kilobase of transcript per million mapped reads (FPKM). (**E**) Predicted transcription factors regulating *PlgSOD* gene expression.

To investigate the evolutionary relationships among PlgSOD proteins, a phylogenetic tree was constructed using the neighbor-joining method. The analysis classified PlgSOD proteins into three major groups based on their structural domains and phylogenetic relationships (Figure 1A). Plant SODs are categorized into Cu/Zn-SOD, Fe-SOD, and Mn-SOD families, according to their metal cofactors [19,20]. The Cu/Zn-SOD family is characterized by the conserved Sod_Cu domain (PF00080) [2], which was detected in PlgSOD4, PlgSOD5, PlgSOD6, and PlgSOD7, confirming their classification as Cu/Zn-SODs (Figure 1C). Notably, PlgSOD4 also contains a heavy metal-associated (HMA) domain (PF00403), a feature shared with Cu/Zn-SOD proteins from other plant species, such as MtCSD2 from *Medicago truncatula* [21], MeCSOD1 from *Manihot esculenta* [20], SbSOD3 from *Sorghum bicolor* [22], SlSOD4 from *Solanum lycopersicum* [23], and CsCSD3 from *Camellia sinensis* [24].

The Fe-SOD (PlgSOD1 and PlgSOD3) and Mn-SOD (PlgSOD2) proteins contain the characteristic iron/manganese superoxide dismutase N-terminal (Sod_Fe_N, PF00081) and C-terminal (Sod_Fe_C, PF02777) domains (Figure 1C), which are essential for Fe- and Mn-SOD functionality [21]. In higher plants, Cu/Zn-SODs are widely distributed in various cellular compartments, while Fe-SODs and Mn-SODs are predominantly localized in the chloroplast and mitochondria, respectively [25]. Consistently, PlgSOD4 to PlgSOD7 were predicted to localize to the chloroplast, whereas PlgSOD2 was identified as mitochondrial. Furthermore, Cu/Zn-SODs typically exhibit acidic pI values, while Fe- and Mn-SODs span a wider range—from acidic to basic—reflecting their functional adaptation to distinct subcellular environments [24]. In line with this, PlgSOD4 to PlgSOD7 exhibited acidic pI values (5.31–6.01), whereas PlgSOD1 to PlgSOD3 displayed a broader range of pI values (Table 1). These findings support the functional classification of PlgSODs and provide insight into their likely roles within specific cellular compartments.

Gene structural diversity is a key driver of evolution in multigene families. To explore the structural organization of *PlgSOD* genes, exon–intron structures were analyzed by aligning cDNA sequences with their corresponding genomic DNA. The number of introns varied from 4 to 8, with *PlgSOD1* containing the highest number (8 introns) and *PlgSOD5* the fewest (4 introns) (Figure 1B), indicating substantial structural variability. Introns were categorized into three phases: phase 0 (54.76%), phase 1 (42.86%), and phase 2 (2.38%). Exon–intron divergence is typically influenced by three primary mechanisms: exon/intron gain or loss, exonization/pseudoexonization, and insertion/deletion events [26]. A comparative analysis of exon–intron structures (Figure 1B) alongside phylogenetic data (Figure 1A) suggests that *PlgSOD4* and *PlgSOD6* originated from a common ancestral gene through duplication, followed by the loss of the first intron in *PlgSOD4*. A similar evolutionary pattern was observed between *PlgSOD1* and *PlgSOD3*, indicating conserved structural modifications. These findings underscore the role of exon–intron remodeling in shaping the evolutionary trajectory of *SOD* genes, as exemplified by the *PlgSOD* family, and highlight the dynamic nature of multigene family diversification.

### 3.2. Analysis of Tissue Expression and Transcriptional Regulation of PlgSODs

Understanding tissue-specific gene expression is essential for elucidating gene function across different plant tissues. In this study, the expression patterns of *PlgSODs* were examined in multiple tissues, including leaves, roots, stems, seeds, petals, pistils, sepals, and stamens. As shown in Figure 1D, *PlgSOD2*, *PlgSOD6*, and *PlgSOD7* exhibited high expression levels across most tissues, whereas *PlgSOD5* showed minimal or undetectable expression. Similarly, *SOD* genes in other plant species also display broad expression across various tissues [20,27,28,29], suggesting their potential involvement in plant growth and development.

To explore potential regulatory interactions between transcription factors (TFs) and *PlgSODs*, the PlantRegMap database (version 5.0) was used, identifying 24 TFs as putative regulators of *PlgSOD* expression. As shown in Figure 1E, most *PlgSOD* genes were predicted to be regulated by TFs from the B3, Dof, and MYB-related families.

The B3 TF family is unique to plants and plays a key role in hormone pathways, particularly those involving auxin, abscisic acid, and brassinosteroids [30]. Dof TFs, also plant-specific, are involved in diverse processes related to growth, development, and responses to biotic and abiotic stresses [31]. MYB-related TFs, a major subgroup of the MYB family, regulate secondary metabolite biosynthesis, plant development, and environmental stress adaptation [32]. The presence of these TF binding sites in *PlgSOD* promoter regions suggests that PlgSODs are likely regulated by complex transcriptional networks integrating hormonal signaling, developmental cues, and stress responses to coordinate physiological and adaptive processes in *P. grandiflorus*.

## 4. Conclusions

This study presents the first comprehensive genome-wide analysis of *SOD* genes in *P. grandiflorus*, identifying seven PlgSODs and classifying them into three major groups based on phylogenetic relationships and conserved domains. *PlgSODs* exhibited significant variation in gene structure, physicochemical properties, and subcellular localization, underscoring their functional diversity. Furthermore, tissue-specific expression patterns revealed distinct contributions of *PlgSOD* genes to various physiological processes. These findings lay the foundation for future functional studies and offer valuable genetic resources for improving stress tolerance in *P. grandiflorus* through molecular breeding and biotechnological approaches.

## Figures and Tables

**Table 1:** Gene catalog and nomenclature of superoxide dismutases (SODs) in *Platycodon grandiflorus*.

Name	Accession Number	Arabidopsis SOD (% Identity)	Gene (bp)	Amino Acids	pI	MW (kDa)	Instability Index	GRAVY	Subcellular Localization
PlgSOD1	Pg_chr01_12590T	AT5G51100 (63%)	3329	298	5.73	33.79	41.98	−0.512	Chloroplast
PlgSOD2	Pg_chr01_13570T	AT3G10920 (80%)	5035	228	7.9	25.25	37.26	−0.341	Mitochondria
PlgSOD3	Pg_chr04_37450T	AT5G23310 (72%)	8936	260	7.72	29.82	38.11	−0.409	Chloroplast
PlgSOD4	Pg_chr04_04620T	AT1G12520 (69%)	4802	321	6.01	33.88	33.77	0.004	Chloroplast
PlgSOD5	Pg_chr05_22460T	AT1G08830 (58%)	4340	284	5.51	31.29	36.81	−0.169	Chloroplast
PlgSOD6	Pg_chr06_29440T	AT2G28190 (72%)	8274	221	5.95	22.35	26.29	0.120	Chloroplast
PlgSOD7	Pg_chr09_16970T	AT1G08830 (80%)	5162	198	5.31	20.48	25.10	0.195	Chloroplast

## Data Availability

The data presented in this study are available on request from the corresponding author. The data are not publicly available due to reasons of privacy.
